# P04 Assessment of antibiotic resistance formation predictors among medical students in developing countries

**DOI:** 10.1093/jacamr/dlad066.008

**Published:** 2023-06-14

**Authors:** Iryna Bondaruk, Kiarina Chichirelo-Konstantynovych, Tetiana Bevz, Yaroslav Demchyshyn

**Affiliations:** National Pirogov Memorial Medical University, Vinnytsya, Ukraine; National Pirogov Memorial Medical University, Vinnytsya, Ukraine; National Pirogov Memorial Medical University, Vinnytsya, Ukraine; National Pirogov Memorial Medical University, Vinnytsya, Ukraine

## Abstract

**Background:**

According to CDC (2019), antibiotic-resistant infections affect more than 2 million people. By 2030, 100 million people will die prematurely as a result of uncontrolled antibiotic use, and in 35 years, this figure will reach 300 million (BSAC, 2018). Despite of modern patient target approach to curing majority of diseases, the problem of antibiotics over usage is still urgent in middle-income countries due to non-profile prescription, absence of microbiological confirmation and the ease of availability of antibiotics.

**Objectives:**

To investigate antibiotic usage expedience for a 1-year duration among Ukrainian and foreign medical students.

**Subjects and methods:**

A total of 150 (Ukrainians 46%, foreigners 54%; aged 18–25 years) medical students were examined by questionnaire for antibiotic usage during last year with specification of reasons, course frequency and duration, expediency of appointment, treatment outcomes, possible reason for cancellation/replacement of antibiotic therapy and preventable usage cases.

**Results:**

The majority of foreigners [60.5% (*n* = 49)] and 33.8% (*n* = 25) Ukrainians used antibiotics for different reasons during February 2022–January 2023 (*P* < 0.01). Antibiotic usage by physician’s prescription was held among 24.49% (*n* = 12) foreign students and 68.18% (*n* = 12) Ukrainian ones (*P* < 0.05). The frequency of antibiotic application due to positive result of bacteriological investigation was significantly lower among foreign responders: 27.27% versus 4.00% in Ukrainians (*P* < 0.05). In the Ukrainian students sample 36.3% (*n* = 8) responders predisposed to antibiotic usage for infectious condition appearance [versus 6.1% (*n* = 3) in the foreign sample, *P* < 0.01]. The frequency of ≥3 antibiotic courses per year prevailed in the foreign student group [12.25% (*n* = 6), *P* < 0.05]. The analysis of post-antibiotic adverse events found out allergic reaction in 1.2% (*n* = 1) in the foreign sample, diarrhoea 1.56% (*n* = 1) and change of antibiotic due to ineffectiveness 1.56% (*n* = 1) in the Ukrainian sample.

**Conclusions:**

Possible predictors of the formation of resistance to antibiotics among young people living in developing countries, such as incorrect clinical use, lack of control over the prescription of antibiotics, ease of availability of antibiotics and as a result of self-medication, were evaluated. The prophylactic prescription of broad-spectrum antibiotics for routine procedures, such as preparation for surgical interventions and in the postoperative period, also draws attention.

